# Clinically Meaningful Improvements in Sperm DNA Fragmentation Severity in Infertile Men Treated with Superoxide Dismutase Supplementation: A Single-Center Experience

**DOI:** 10.3390/jcm11216540

**Published:** 2022-11-04

**Authors:** Luca Boeri, Gianpaolo Lucignani, Letizia Maria Ippolita Jannello, Matteo Turetti, Irene Fulgheri, Carlo Silvani, Franco Gadda, Paola Viganò, Edgardo Somigliana, Emanuele Montanari

**Affiliations:** 1 Department of Urology, Foundation IRCCS Ca’ Granda—Ospedale Maggiore Policlinico, 20122 Milan, Italy; 2 Department of Vascular Surgery, Foundation IRCCS Ca’ Granda—Ospedale Maggiore Policlinico, 20122 Milan, Italy; 3 Infertility Unit, Foundation IRCCS Ca’ Granda—Ospedale Maggiore Policlinico, 20122 Milan, Italy; 4 Department of Clinical Sciences and Community Health, University of Milan, 20122 Milan, Italy

**Keywords:** male infertility, sperm DNA fragmentation, superoxide dismutase, antioxidant, semen parameters

## Abstract

Background. Antioxidants are commonly used for the treatment of idiopathic male infertility. Previous studies have shown that antioxidants are able to improve sperm quality, but little is known about their impact on sperm DNA fragmentation (SDF). Preliminary findings showed that superoxide-dismutase (SOD)-based antioxidant plus hydroxytyrosol and carnosol (FertiPlus^®^ SOD) therapy was associated with SDF improvement in a small cohort of infertile men. Therefore, we aimed to assess rates of and predictors of semen parameters and SDF improvements in infertile men treated with FertiPlus^®^ SOD therapy (SOD+) or with other antioxidants without SOD (SOD−) in the real-life setting. Methods. Data from 60 consecutive infertile men with baseline SDF ≥ 30% and treated with SOD+ or SOD− for at least three months were analyzed. Clinical parameters and serum hormones were collected. Sperm parameters and SDF were requested at baseline and after SOD+ or SOD− treatment. Clinically meaningful SDF change after treatment was defined as SDF improvement >20% compared to baseline. Propensity score matching was performed to adjust for baseline differences between groups. Descriptive statistics were used to compare clinical and hormonal characteristics between SOD+ and SOD− groups. Semen characteristics were compared before and after treatment. Logistic regression models investigated the association between clinical variables and SDF improvement. Results. Groups were similar in terms of clinical, serum hormones and semen parameters at baseline after matching. Compared to baseline, sperm progressive motility (17 (10–36)% vs. 27 (12–41)%) and normal morphology (2 (1–6)% vs. 4 (2–6)%) significantly improved after SOD+ treatment (all *p* < 0.01), but not after SOD−. SDF values significantly improved after treatment in both groups, compared to the baseline evaluation (all *p* < 0.01). However, SDF values were lower after SOD+ than SOD− treatment (30 (22–36)% vs. 37 (31–42)%, *p* = 0.01). Similarly, a clinically meaningful improvement in SDF at follow-up was more frequently found after SOD+ than SOD− treatment (76.7% vs. 20.0%, *p* = 0.001). Multivariable logistic regression analysis showed that SOD+ treatment (OR 5.4, *p* < 0.001) was an independent predictor of clinically meaningful SDF improvement, after accounting for age and baseline FSH values. Conclusions. This cross-sectional study showed that, in a cohort of primary infertile men with SDF ≥ 30%, SOD-based treatment was significantly effective in improving SDF compared to antioxidants without SOD. Approximately 80% of men treated with SOD+ achieved clinically meaningful improvement in SDF after three months of treatment. Sperm progressive motility and normal morphology also improved after SOD+ therapy but not after SOD−. These results suggest that SOD+ treatment could be considered an effective option for the management of idiopathic infertile men with elevated SDF.

## 1. Introduction

Infertility is a common disease in Western countries that affects approximately 15% of couples of reproductive age [1]. In this context, male factor infertility (MFI) can be identified in about half of the cases. Several causes of MFI have been identified, including hormonal disorders, recreational habits [2,3], systemic diseases [4,5], urogenital infections [6,7] and genetic disorders [8,9]. However, infertility is still idiopathic in nature in about 30% of cases. Therefore, current guidelines mandate a focused diagnostic work-up of both partners of infertile couples to identify the exact cause of infertility [1,10,11,12]. For males, this should include a medical and reproductive history, a physical examination, hormonal investigation and semen analysis, with adherence to World Health Organization (WHO) reference values [13]. Furthermore, the comprehensive medical investigation of infertile males acquires even more importance in terms of health preservation when considering that poor sperm quality, and MFI per se, have been associated with overall health in males and the risk of developing further comorbid disease later in life [14,15].

Several non-invasive treatments are available for idiopathic male infertility. Among these, antioxidants are the most commonly used in clinical practice [1,12]. Oxidative stress (OS) is considered to be one of the most important contributing factors in the pathogenesis of idiopathic infertility. Reactive oxygen species, the final products of OS, can impair sperm function by acting at several levels, including plasma membrane lipid peroxidation, which can affect sperm motility, the acrosome reaction and chromatin maturation, leading to increased DNA fragmentation [16,17]. In this context, sperm DNA fragmentation (SDF) has progressively gained clinical importance in terms of reproductive outcomes both under natural and assisted reproductive technology (ART) conditions [18]. Antioxidants are substances that neutralize or protect the cells against the detrimental effects of oxidation and free radicals. The antioxidant system has enzymatic or non-enzymatic factors. Enzymatic antioxidants include superoxide dismutase (SOD), catalase, glutathione peroxidase and glutathione reductase. Non-enzymatic ones include glutathione, cysteine, N-acetylcysteine (NAC), carotenoids, vitamin C, vitamin E, carnitine, ferritin, L-arginine, transferrin, Coenzyme Q10, myo-inositol, lycopene, selenium, zinc and folate [19]. Free-radical scavenging, neutralization and preserving sperm DNA integrity and mitochondrial transport are the most commonly recognized mechanisms of antioxidant action [20]. A previously published systematic review reported that vitamin E, vitamin C, NAC, carnitines, Coenzyme Q10, lycopene, selenium and zinc were associated with improved sperm concentration, motility and morphology [20]. Similarly, supplementation with NAC, Coenzyme Q10 and vitamin C and E resulted in significant improvement in SDF in infertile men [20].

Among antioxidant systems, the one mediated by the nuclear factor [erythroid-derived 2]-like 2 transcription factor (Nrf2) was found to be one of the most efficient pathways, being able to counteract oxidative stress by stimulating the production of antioxidant substances [21]. Only a few studies have investigated the effectiveness of SOD-based antioxidant therapy plus hydroxytyrosol and carnosol (FertiPlus^®^ SOD), which is known to activate the Nrf2 system, in improving conventional sperm parameters and in reducing SDF in idiopathic infertile men [21,22].

Therefore, we performed a cross-sectional study assessing rates and predictors of semen parameters and SDF improvement in idiopathic infertile men treated with either SOD-based antioxidant plus hydroxytyrosol and carnosol (FertiPlus^®^ SOD) therapy or with other antioxidants without SOD, hydroxytyrosol and carnosol in the real-life setting.

## 2. Materials and Methods

We retrospectively analyzed data from a cohort of 148 white European men consecutively assessed at a single academic center for primary couple infertility between January 2019 and September 2022. Data were prospectively collected and, for the specific purpose of this study, we only considered patients retrospectively assigned to one of two groups based on the treatment they had undergone: SOD+ (SOD-based antioxidant plus hydroxytyrosol and carnosol; FertiPlus^®^ SOD) or SOD− (any other antioxidants without SOD, hydroxytyrosol and carnosol) for no less than 3 months. Of note, SOD− compounds included a combination of vitamin C, vitamins B, Coenzyme Q10, myo-inositol, selenium and zinc. The choice of the treatment was decided based on patient and physician preference.

According to the WHO criteria, infertility is defined as not conceiving a pregnancy after at least 12 months of unprotected intercourse regardless of whether or not a pregnancy ultimately occurs [23]. Patients were included if they were ≥18 and ≤50 years old and had pure MFI, defined after a comprehensive diagnostic evaluation of all the female partners.

All participants were homogenously assessed by the same expert academic urologist (L.B.) with a thorough medical history and a complete physical examination. Health-significant comorbidities were scored with the Charlson Comorbidity Index (CCI), coded using the International Classification of Diseases, 9th revision [24,25]. Likewise, weight and height were measured, calculating body mass index (BMI) for each participant [26]. Testes volume (TV) was assessed in all cases using Prader’s orchidometer estimation [27]; for the specific purpose of this study, we calculated the mean value between the two sides. Varicocele was also clinically assessed in every patient.

Venous blood samples were drawn from each patient between 7 AM and 11 AM after an overnight fast. Follicle-stimulating hormone (FSH), luteinizing hormone (LH), total testosterone (tT), prolactin and thyroid-stimulating hormone (TSH) levels were measured for every individual. According to our internal diagnostic protocol, chromosomal analysis and genetic testing were performed in every infertile man (i.e., karyotype analysis and Y-chromosome microdeletion and cystic fibrosis mutation tests) [28].

At baseline, all patients underwent two consecutive semen analyses at least 3 months apart [29]; semen samples were collected by masturbation after a sexual abstinence of 2–7 days and analyzed within 2 h of ejaculation, in accordance with the WHO criteria. For the specific purposes of this study, we considered semen volume, sperm concentration, progressive sperm motility and normal morphology. Before treatment, the SDF index was measured by a flow cytometric analysis according to the sperm chromatin structure assay (SCSA^®^) [30]. The same laboratory was used for analyses of all parameters.

The assessment visit was performed in-person after a minimum of 3 months of SOD+ or SOD− treatment. At follow-up, semen parameters and SDF were recorded. As previously reported, we considered an SDF improvement >20% after treatment as clinically meaningful [22].

The primary endpoint of the study was to assess the proportion of infertile men who: (i) achieved SDF improvement > 20% (vs. baseline) and (ii) showed SDF < 30% (returned to normal values) after SOD+ versus SOD− therapy. After treatment, we also evaluated the change from baseline to follow-up in terms of conventional semen characteristics and the difference in sperm parameters between groups.

Patients were retrospectively included in the study if they had pure MFI and had SDF ≥ 30% before treatment [12]. Exclusion criteria were: symptoms suggestive of genitourinary infections; a history of vasectomy, undescended testicle, hypospadias or infertility treatment in the preceding year; and partial or incomplete data concerning one or more of the semen parameters considered.

A convenient sample of 92 infertile men treated with SOD+ (*n* = 41) and SOD− (*n* = 52) were considered for the final statistical analyses.

Data collection followed the principles outlined in the Declaration of Helsinki. All men signed their informed consent agreeing to share their own anonymous information for future studies. The study was approved by our Hospital Ethical Committee (Prot. 2021—ESQLFDI).

### Statistical Methods

#### Sample Size Calculation

The sample size consisted of 25 patients in each group, calculated using the two-sample *t*-test analysis. A preview study showed that the mean (standard deviation) SDF improvement after SOD+ vs. SOD− therapy was 21 (24)% [22]. Therefore, we considered a difference in means of 21% and a variability (sigma) of 24%. Considering alpha = 0.05 and beta = 0.20 (power = 1 − beta = 0.8), at least 25 patients were needed to obtain a power of 80% (Russ Lenth applet for Windows). Considering a 10% lost-to-follow-up rate, we included 30 patients in each group.

Distribution of data was tested with the Shapiro–Wilk test. Data are presented as medians (interquartile range; IQR) or frequencies (proportions). In order to control for measurable baseline differences among patients in the two groups, we relied on propensity-score-matched (PSM) analyses to adjust for those differences [31]. Propensity scores were computed by modeling logistic regression with the odds of receiving SOD+ therapy as the dependent variable and age, BMI, TV and sperm concentration as the independent variables. Subsequently, the SOD+ and SOD− groups were matched using the propensity score (1:1 nearest-neighbor PSM analyses using a caliper width of 0.2 of the standard deviation of the logit of the propensity score).

The analyses consisted of several statistical steps. First, baseline clinical, laboratory parameters and semen characteristics were compared between SOD+ and SOD− groups with the Mann–Whitney test and the chi-square test. Second, the paired *t*-test assessed potential differences in sperm parameters and SDF values at the 3-month follow-up assessment compared to baseline among both groups. At follow-up, semen parameters were compared between groups with the Mann–Whitney test, while the proportion of participants who achieved an SDF improvement > 20% or returned to normal values (SDF < 30%) were compared between SOD+ and SOD− treatments with the chi-square test. Finally, univariable (UVA) and multivariable (MVA) logistic regression analyses tested the associations between study variables and >20% SDF improvement after treatment.

Statistical analyses were performed using SPSS v.26 (IBM Corp., Armonk, NY, USA). All tests were two-sided, and statistical significance was determined at *p* < 0.05.

## 3. Results

Table 1 details clinical, hormonal and semen characteristics of infertile men submitted to SOD+ and SOD− treatment. Overall, median (IQR) age and BMI were 36 (32–39) years and 25.2 (23.7–27.1) kg/m^2^, respectively. Median TV was 18 (14–20) while median tT was 4.6 (3.5–5.9) ng/mL. Groups were similar in terms of clinical characteristics and recreational habits. Serum hormones were comparable among patients treated with SOD+ and SOD−. After matching, sperm concentration, progressive motility and normal morphology were similar between groups. Baseline median SDF was 41 (30–53)% in the whole cohort, with no differences according to SOD treatment.

Table 2 reports semen characteristics at the 3-month follow-up assessment. As expected, semen volume remained stable after treatment in both groups. Similarly, sperm concentration slightly increased, but without any statistical differences compared to baseline. Moreover, sperm concentration did not differ according to the treatment used. Compared to baseline, sperm progressive motility significantly improved after 3 months of SOD+ treatment (*p* < 0.01). This was not the case in the SOD− group. Furthermore, at follow-up assessment, SOD+ patients had higher sperm progressive motility than those treated with SOD− (27 (12–41)% vs. 20 (9–39)%, *p* = 0.01). The same was noted for normal sperm morphology. After SOD+ treatment, sperm morphology significantly improved compared to baseline (*p* < 0.01). At follow-up, normal sperm morphology was higher in SOD+ patients than in SOD− patients (4 (2–6)% vs. 2 (2–4)%, *p* = 0.02). SDF values significantly improved after treatment in both groups compared to baseline evaluation (all *p* < 0.01). However, SDF values were lower after SOD+ compared to SOD− treatment (30 (22–36)% vs. 37 (31–42)%, *p* = 0.01). A higher proportion of participants in the SOD+ group returned to normal SDF values after treatment, compared to those in the SOD− group (36.6% vs. 13.3%, *p* = 0.03). Similarly, an SDF improvement >20% at follow-up was more frequently found after SOD+ than SOD− treatment (76.7% vs. 20.0%, *p* = 0.001).

Table 3 reports the logistic regression model predicting SDF improvement >20% at follow-up. Univariable analysis revealed that younger age (OR 0.8, *p* = 0.01) and lower FSH values (OR 0.7, *p* < 0.01) at baseline, along with SOD+ treatment (OR 8.5, *p* < 0.001), were all associated with SDF improvement >20%. Multivariable logistic regression analysis confirmed that SOD+ treatment (OR 5.4, *p* < 0.001) was an independent predictor of SDF improvement >20%, after accounting for age and baseline FSH values.

## 4. Discussion

This study was specifically designed to evaluate the impact of SOD-based antioxidant plus hydroxytyrosol and carnosol (FertiPlus^®^ SOD) therapy on conventional semen parameters and SDF values in a cohort of idiopathic infertile men. We found that SOD+ treatment significantly improved sperm motility, normal sperm morphology and SDF, as compared to the baseline evaluation. Of clinical importance, SOD+ therapy was more effective than SOD− in improving sperm parameters and SDF after three months of treatment.

Antioxidants are commonly used in clinical practice for the treatment of idiopathic male infertility because of their safety profile and effectiveness [12,20]. Several molecular mechanisms of antioxidants have been associated with semen parameter improvement, including free-radical scavenging, neutralization and preserving sperm DNA integrity and mitochondrial transport [20]. Sperm DNA integrity has been recently recognized as an important prognostic index for couple infertility, being associated with natural and ART-related outcomes, semen quality and recurrent pregnancy loss [18]. SDF is primary induced by defective maturation and abortive apoptosis occurring within the testis or by oxygen species throughout the male reproductive tract [32]. Lifestyle and recreational habits, systemic inflammation and environmental pollution are all generators of oxidative stress, which is responsible for increasing SDF in infertile men [17]. Antioxidants have been investigated as potential treatment for SDF, but results are inconclusive [20] mainly due to the heterogeneity of the published studies in terms of the compound used and timing of treatment.

The Nrf2 pathway has been proved to have effective antioxidant capacities with potential clinical implications in terms of male infertility treatment [22]. Negri et al. retrospectively analyzed infertile men treated with SOD+ (*n* = 55) or other antioxidants (*n* = 48) for two months. They found that semen parameters did not change, but SDF improved only after SOD treatment [22]. In our study, conducted in primary infertile men with pathologic SDF at baseline, we found that SOD+ treatment for at least three months significantly improved sperm motility and normal sperm morphology as compared to SOD− treatment. Furthermore, one out of three infertile men returned to normal SDF values and approximately 80% of participants achieved SDF improvement > 20% (considered clinically relevant) after SOD+ therapy. This was not the case for patients treated with SOD− therapy. Patients who underwent SOD+ therapy had a 5-fold higher chance of clinically meaningful improvement in SDF than those treated with SOD− even after adjusting for age and FSH values at baseline. Overall, these findings confirm that SOD-based therapy might have a relevant role in the treatment of idiopathic infertile men with elevated SDF values.

Our study has several strengths. First, we investigated a cohort of the same ethnicity: white European primary infertile men with an identical thorough clinical, hormonal and semen evaluation. Treatment protocol and follow-up were standardized as managed by the same expert in reproductive medicine. Second, we relied on PSM to adjust for potential baseline confounders between groups, which is a classic bias of retrospective investigations. Third, we included only men with pathologic SDF values at baseline, thus showing that SOD+ treatment is effective even in infertile men with “severe” semen impairment. Overall, we provide novel evidence showing that one out of three infertile men with pathologic SDF values could achieve normalization after three months of SOD-based therapy, but this was not noted in patients treated with other antioxidants. Moreover, 80% of infertile men treated with SOD+ had a clinically meaningful improvement in SDF at follow-up, thus confirming that FertiPlus^®^ SOD is an effective option for reducing SDF. Therefore, our results achieved important clinical focus in real-life setting in terms of patient management and expectations.

Our study is not devoid of limitations. First, despite the rigorous methodology, this was a single center-based study, raising the possibility of selection biases; therefore, larger studies across different center and cohorts are needed to externally validate our findings. Second, we could not provide longer follow-up data in terms of semen characteristics; however, 3 months of antioxidant treatment is a standardized duration in clinical practice [12,20]. Lastly, serum hormones were not requested at follow-up; therefore, we were unable to investigate the potential impact of SOD treatment on testosterone and gonadotropin values.

## 5. Conclusions

In this cross-sectional study, we found that approximately 80% of infertile men achieved a clinically meaningful improvement in SDF after three months of SOD-based antioxidant plus hydroxytyrosol and carnosol (FertiPlus^®^ SOD) treatment. Moreover, one out of three infertile men returned to normal SDF values after SOD+ treatment. Sperm progressive motility, normal morphology and SDF significantly improved after SOD+ therapy, but this was not the case for patients treated with other antioxidants without SOD. In light of the recognized negative impact of elevated SDF on natural and assisted pregnancy outcomes, SOD+ treatment emerged as an important option for the management of idiopathic infertile men.

## Figures and Tables

**Table 1:** Baseline characteristics of the whole cohort of patients (*n* = 60).

	Overall	SOD+	SOD−	*p*-Value *
No. of individuals	60	30 (50.0%)	30 (59.0%)	
Age (years)				0.6
Median (IQR)	36 (32–39)	36 (32–41)	36 (32–40)	
Range	18–50	18–50	25–50	
Duration of infertility (months)				0.3
Median (IQR)	24 (12–38)	24 (12–38)	24 (15–40)	
Range	12–200	12–200	24–108	
BMI (kg/m^2^)				0.4
Median (IQR)	25.2 (23.7–27.1)	25.1 (23.8–26.3)	25.2 (23.8–28.7)	
Range	18.9–41.0	18.9–41.0	20.9–41.0	
CCI (score)				0.8
Median (IQR)	0.0 (0.0)	0.0 (0.0)	0.0 (0.0)	
Mean (SD)	0.1 (0.2)	0.1 (0.1)	0.1 (0.1)	
Range	0–4	0–1	0–4	
Current smoking status (No. (%))	27 (45.0)	13 (43.3)	14 (46.6)	0.6
Mean TV (Prader’s estimation)				0.7
Median (IQR)	18 (14–20)	18 (18–25)	18 (14–25)	
Range	5–25	5–25	8–25	
Varicocele (No. (%))	21 (35.0)	10 (33.3)	11 (36.6)	0.2
tT (ng/mL)				0.7
Median (IQR)	4.6 (3.5–5.9)	4.5 (3.1–6.1)	4.6 (3.7–6.5)	
Range	0.9–21.5	0.9–11.9	2.0–21.5	
FSH (mUI/mL)				0.5
Median (IQR)	6.4 (3.0–9.5)	6.5 (3.1–8.8)	6.4 (2.9–9.1)	
Range	0.8–45.8	2.1–15.3	0.8–45.8	
LH (mUI/mL)				0.3
Median (IQR)	5.2 (3.1–7.2)	5.1 (3.3–7.5)	5.2 (3.5–8.4)	
Range	1.9–34.2	1.9–13.7	3.1–34.2	
Prolactin (ng/mL)				0.8
Median (IQR)	7.9 (5.1–11.5)	7.8 (4.9–10.7)	8.1 (5.6–11.9)	
Range	1.9–24.3	1.9–24.3	2.6–23.9	
TSH (mUI/L)				0.5
Median (IQR)	1.5 (1.2–2.7)	1.5 (1.1–2.7)	1.6 (1.2–2.1)	
Range	0.9–4.6	0.9–4.2	0.9–4.6	
Sexual abstinence (days)				0.9
Median (IQR)	3 (2–5)	3 (2–5)	3 (2–5)	
Range	2–7	2–7	2–7	
Semen volume (mL)				0.8
Median (IQR)	3.0 (2.0–4.0)	3.0 (2.0–4.5)	3.0 (2.0–4.0)	
Range	1.0–11.0	1.0–11.0	1.0–9.0	
Sperm concentration (×10^6^/mL)				0.5
Median (IQR)	16.3 (4.9–23.1)	16.1 (3.1–19.3)	16.2 (4.6–24.6)	
Range	0.9–25.8	0.9–24.7	1.1–25.8	
Progressive sperm motility (%)				0.7
Median (IQR)	16 (10–39)	17 (10–36)	16 (9–31)	
Range	0–46	0–46	0–45	
Normal sperm morphology (%)				0.4
Median (IQR)	2 (1–6)	2 (1–6)	2 (1–6)	
Range	0–10	0–10	0–10	
SDF (%)				0.8
Median (IQR)	41.0 (30–53)	41.2 (30–51)	40.7 (30–55)	
Range	30–97	30–86	30–97	

Keys: BMI = body mass index; CCI = Charlson Comorbidity Index; TV = testicular volume; tT = total testosterone; FSH = follicle-stimulating hormone; LH = luteinizing hormone; TSH = thyroid-stimulating hormone; SDF = sperm DNA fragmentation index. * *p*-value according to the Mann–Whitney test and chi-square test, as indicated.

**Table 2:** Semen characteristics at 3-month follow-up assessment.

	SOD+	SOD−	*p*-Value *
Semen volume (mL)			0.9
Median (IQR)	3.0 (2.0–4.0)	3.0 (2.0–4.0)	
Range	1.0–10.0	1.0–9.0	
Sperm concentration (×10^6^/mL)			0.2
Median (IQR)	20.2 (5.4–25.1)	18.9 (4.9–24.1)	
Range	1.1–34.6	0.9–29.9	
Progressive sperm motility (%)			0.01
Median (IQR)	27 (12–41) ^§^	20 (9–39)	
Range	0–50	0–45	
Normal sperm morphology (%)			0.02
Median (IQR)	4 (2–6) ^§^	2 (2–4)	
Range	0–10	0–10	
SDF (%)			0.01
Median (IQR)	30 (22–34) ^§^	37 (31–42) ^§^	
Range	10–75	15–84	
SDF < 30% (No. (%))	11 (36.6)	4 (13.3)	0.03
SDF improvement > 20% (No. (%))	23 (76.7)	6 (20.0)	0.001

Keys: SDF = sperm DNA fragmentation index; * *p*-value according to unpaired Mann–Whitney test and chi-square test, as indicated. ^§^ *p* < 0.01 vs. baseline. *p*-value according to paired *t*-test.

**Table 3:** Logistic regression models predicting SDF improvement >20% in the whole cohort (no. = 60).

	UVA Model			MVA Model		
	OR	*p*-Value	95% CI	OR	*p*-Value	95% CI
Age	0.85	0.01	0.81–0.95	0.86	0.01	0.79–0.92
CCI	1.01	0.5	0.87–3.12			
TV	1.15	0.07	0.94–2.76			
FSH	0.78	<0.01	0.71–0.89	0.82	<0.01	0.76–0.94
SOD+ treatment	8.49	<0.001	3.97–12.99	5.41	<0.001	2.91–12.78

Keys: UVA = univariate model; MVA = multivariate mode; CCI = Charlson Comorbidity Index; TV = testicular volume; FSH = follicle-stimulating hormone; SOD = superoxide dismutase.

## Data Availability

Data available on request due to restrictions.
